# Photocatalytic Activity of Silicon Nanowires Decorated with PbS Nanoparticles Deposited by Pulsed Laser Deposition for Efficient Wastewater Treatment

**DOI:** 10.3390/ma15144970

**Published:** 2022-07-17

**Authors:** Faisal K. Algethami, Khaled Trabelsi, Anouar Hajjaji, Mohamed B. Rabha, Lotfi Khezami, Mohamed R. Elamin, Brahim Bessais, My Ali El Khakani

**Affiliations:** 1 Chemistry Department, College of Science, Imam Mohammad Ibn Saud Islamic University (IMSIU), P.O. Box 90905, Riyadh 11623, Saudi Arabia; falgethami@imamu.edu.sa (F.K.A.); lhmkhezami@imamu.edu.sa (L.K.); mrdoshine@yahoo.fr (M.R.E.); 2 Laboratoire de Photovoltaïque, Centre de Recherches et des Technologies de l’Energie, Technopôle de Borj-Cédria, BP 95 Hammam-Lif, Tunis 2050, Tunisia; khaled0984@hotmail.com (K.T.); physicshajjaji@gmail.com (A.H.); brahim.bessais@crten.rnrt.tn (B.B.); 3 Laboratoire de Nanomatériaux et Systèmes pour Energies Renouvelables, Centre de Recherches et des Technologies de l’Energie, Technopôle de Borj-Cédria, BP 95 Hammam-Lif, Tunis 2050, Tunisia; 4 Centre Énergie Matériaux et Télécommunications (INRS-EMT), Institut National de la Recherche Scientifique (INRS), 1650 Boulevard Lionel Boulet, Varennes, QC J3X 1S2, Canada; m.a.elkhakani@inrs.ca

**Keywords:** silicon nanowires, PbS nanoparticles, pulsed laser deposition, photocatalytic degradation

## Abstract

The present work aims to study the photocatalytic properties of nanohybrids composed of silicon nanowires (SiNWs) decorated with PbS nanoparticles (NPs). The elaborated material was intended to be utilized in wastewater treatment. The SiNWs were elaborated from the Metal Assisted Chemical Etching route (MACE), while the PbS NPs were deposited at room temperature onto SiNWs using the pulsed laser deposition (PLD) technique. The influence of decorating SiNWs (having different lengths) with PbS-NPs on their structural, morphological, optoelectronic, and photocatalytic properties was scrutinized. PbS/SiNWs nanohybrids exhibited enhanced photocatalytic degradation towards Black Amido (BA) dye for 20 µm SiNWs length and 0.2% of BA volume concentration. These optimized conditions may insinuate that this nanocomposite-like structure is a promising efficient photocatalytic systems contender, cost-effective, and recyclable for organic compound purification from wastewaters.

## 1. Introduction

The first 1D nanostructures synthesized were carbon nanotubes, discovered in 1991 by Iijima [1]. Following this singular discovery, researchers became interested in synthesizing and characterizing other 1D structures, including nano-sticks, nanoribbons, and nanowires (NWs). Silicon nanowires (SiNWs) were discovered in 1964 by chemical vapor deposition (CVD) of Si on gold aggregates [2]. Silicon nanowires play an essential role in the race for miniaturization of future electronic components, allowing a higher integration density, which would multiply the functions of devices. These applications concern a wide field of science and technology. SiNWs entice global investigation concern owing to their attractive characteristics and usage in various domains, i.e., electronics, diagnostics, solar cells, photovoltaics, catalysis, batteries, analytical chemistry, molecular sensing, etc. [3,4,5,6,7,8,9,10,11]. The SiNWs were elaborated by the Ag-assisted chemical etching technique (Ag-ACE) [12], and the PbS nanoparticles were deposited by pulsed laser deposition [13,14,15]. On the other hand, semiconductor-mediated photocatalysis is a widely adopted procedure [16,17,18,19,20] to degrade dyes and organic contaminants in wastewater, especially those decorated with nanoparticles [21,22]. It is our belief that the photodegradation effectiveness of dyes by PbS/SiNWs nanohybrids is reported first in this work. Amido Black is preferred as a typical dye molecule because it is usually utilized in staining proteins and is highly toxic. The present study evaluated the photocatalytic performance of the PbS/SiNWs nanohybrids by changing the SiNWs lengths and exposure time to UV irradiation for a fixed and varied dye concentration. Scanning Electron Microscopy (SEM, TESCAN VEGA3) and energy-dispersive X-ray spectroscopy (EDS) were accomplished to examine the nanostructured morphology and the elemental analysis of the PbS/SiNWs nanohybrids. TEM images were conveyed with an FEI Tecnai G20 microscope working at 200 kV and supplied with a LaB6 filament. Reflectivity measurements and spectral absorption were performed using a Perkin-Elmer Lambda 950 UV-visible-NIR spectrophotometer. A WCT-120 Silicon Wafer Lifetime Tester was exploited to consider the transient photoconductance and derive the carrier minority lifetime. Ultimately, a photochemical reactor equipped with a 15 W ultraviolet lamp was used for photocatalysis operation.

## 2. Elaboration of Silicon Nanowires Decorated with PbS Nanoparticles

### 2.1. Silicon Cleaning

In all our experimental studies, we used 1 × 1 cm^2^ (100) oriented boron-doped Si wafers having a resistivity of 0.01–0.02 Ω.cm and a thickness of 450 μm. Each substrate undergoes a preliminary chemical cleaning called “CP_4_ chemical pickling”. During this step, the substrates are first immersed for one minute in a solution composed of nitric acid, hydrofluoric acid, and acetic acid (64% HNO_3_, 16% HF, and 20% CH_3_COOH) to remove the surface layer, which contains a high density of structural defects caused by the cutting. The samples are then rinsed thoroughly with deionized water and dried. The CP_4_ solution allows anisotropic etching of Si. Indeed, this acid mixture attacks all crystallographic directions at the same speed. The reaction mechanism of this etching starts with the oxidation of Si by nitric acid (HNO_3_); the fluoride ions coming from the dissociation of HF react with Si to produce water-soluble hexafluoro-silicic acid (H_2_SiF_6_). 

### 2.2. Elaboration of Silicon Nanowires

The metal-assisted etching technique requires the deposition of metallic NPs on the previously cleaned Si substrates [23,24]. The choice of the metallic NPs (Ag, Pt, Au, etc.) and the control of the deposition parameters (deposition time and temperature, concentration of the catalytic solution) are crucial in determining the morphology of the Si nanostructures formed after the etching step. In this work, silver nitrate (AgNO_3_) was dissociated in an aqueous HF solution to provide Ag+ ions according to the following total reaction:

AgNO_3_ → Ag^+^ + NO_3_ (1)

**Figure 1:**
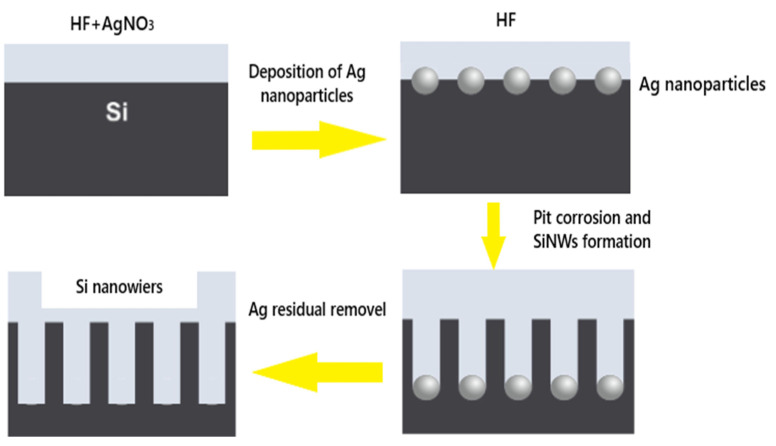
Scheme of the Ag-ACE experimental set-up.

The Ag^+^ ions are reduced to metallic Ag and spontaneously deposit on the Si surface without any electrical energy input by this Electroless Metal Deposition (EMD) method. After an optimization study, the cleaned Si substrates are immersed, during the first step, in an aqueous solution of HF and AgNO_3_. The Ag deposition time, the solution temperature, and the HF/AgNO_3_ concentration are fixed at 1 min, 25 °C, and 4.8/0.02 M, respectively. To achieve the SiNWs, the Ag-NPs covered Si substrates are dipped in a polypropylene Teflon beaker containing an (HF/H_2_O_2_: 4.8/0.2 M) solution. This immersion enabled us to achieve quasi-vertical grooves; it was performed for four different durations (20 min, 30 min, 40 min, and 50 min) to produce various SiNWs lengths. Figure 1 illustrates the experimental setup of the Ag-ACE technique.

### 2.3. Silicon Nanowires Decorated with PbS Nanoparticles Deposited by PLD

**Figure 2:**
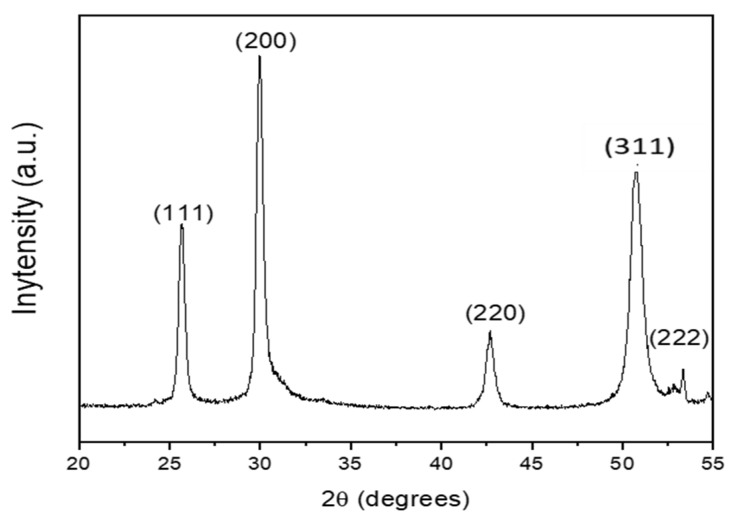
XRD diagram of PbS crystals PLD-deposited at room temperature on intrinsic (100) oriented silicon.

The PLD of PbS films was performed by using a KrF excimer laser (wavelength = 248 nm, pulse duration = 14 ns, repetition rate = 20 Hz, pulse energy = 120 mJ, and laser spot area = 3.4 mm^2^), which was focused on a rotating PbS pellet at an incident angle of 45°. It interacted with a target and produced ejected species with very high kinetic energy. The laser was focused by a lens (focal length: 75 cm) on the target at a frequency of 20 Hz. The deposition of the PbS NPs was performed at room temperature on SiNWs and a quartz substrate. The substrates were placed on a rotating substrate holder (to ensure uniform surface ablation) parallel to the target at 7.9 cm from it. Before ablation, the deposition chamber was pumped under air vacuum to 510^−6^ Torr by a molecular turbopump, under a He pressure of 50 mTorr. The deposition rate was about 0.03 nm/pulse. The fixed number of laser pulses was NLp = 1000 so that the effective assumed total thickness of PbS was about 30 nm. Figure 2 shows the XRD patterns of PLD-PbS films deposited on intrinsic Si substrates under a He pressure of 50 mTorr. The XRD peaks are annotated owing to the standard XRD peaks associated to the crystallographic plans of PbS powder (JCPDS card N° 05-592). First, all the XRD peaks agree with the fingerprint of PbS, confirming the polycrystalline nature of the films even if they were PLD-deposited at room-temperature. Second, one can notice that at a background He pressure of 50 mTorr, the PLD-PbS films exhibit a preferential (200) orientation. It was previously demonstrated that the preferential orientation depends on the He background pressure [13]. In fact, the highly energetic aspect of the laser ablated species provides a higher surface diffusivity to atoms, which would favor the crystallization of PbS without any additional heating of the substrate. On the other hand, no other peaks associated with PbO, PbO_2_, PbSO_3_, PbSO_4_, etc. were observed, confirming thereby the pure PbS cubic phase of the films.

### 2.4. Microstructure and Morphology of PbS/SiNWs Nanohybrids

**Figure 3:**
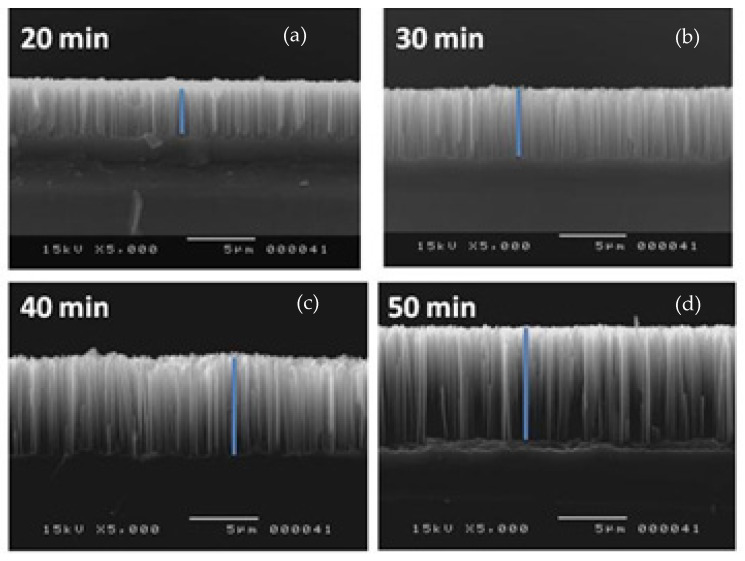
Cross-sectional SEM images showing SiNWs on which 30 nm PbS films were deposited for different etching times (**a**) 20 min, (**b**) 30 min, (**c**) 40 min, (**d**) 50 min.

Figure 3 shows that silicon nanowires (SiNWs) decorated with PbS nanoparticles (etch time between 20–50 min) are vertically aligned. 

**Figure 4:**
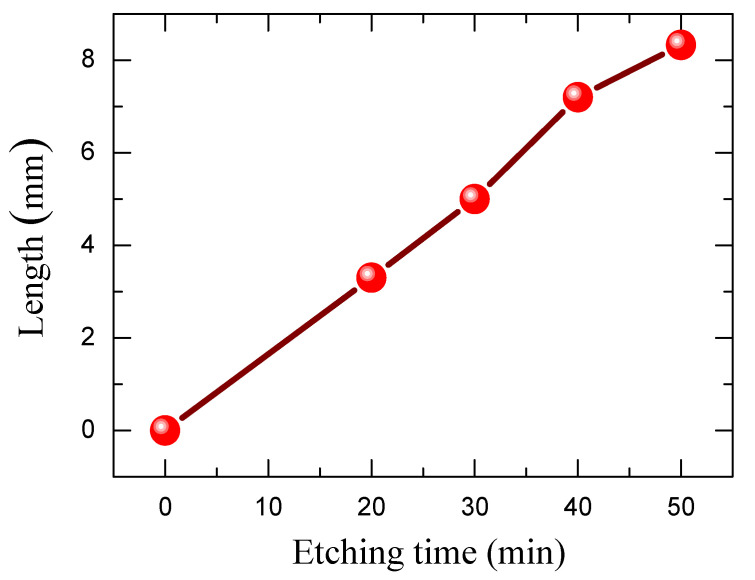
Length of Si NWs as a function of Ag-assisted chemical etching time.

Obviously, the etching time variation from 20 to 50 min results in a gradual increase in the NWs length from approximately 3.3 μm to 8.3 μm. Figure 4 illustrates the length of the SiNWs as a function of etching time. It is worth noting that the Si dissolution regime follows a linear trend for an etching time in the range of 20–50 min.

**Figure 5:**
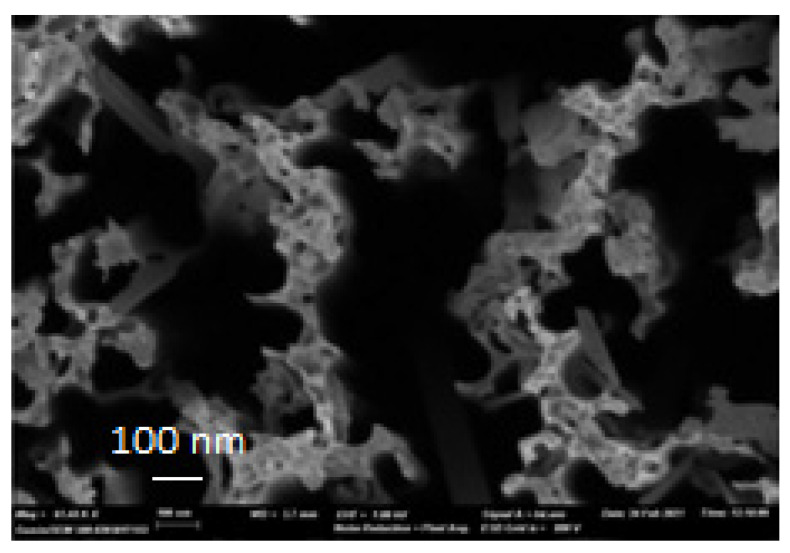
SEM surface image of 20 min MACE SiNWs decorated with PbS NPs.

Figure 5 depicts a magnified SEM surface image of the PbS/SiNWs nanohybrids and the corresponding EDX spectrum. The elements Pb, S, Ag, Si, and O were detected, confirming the purity of the PbS films. However, the SiNWs still contain Ag residue even after Ag etching at the end of the SiNWs formation (Table 1). 

### 2.5. Optoelectronic and Microstructural Properties of PbS/SiNWs Nanohybrids

**Figure 6:**
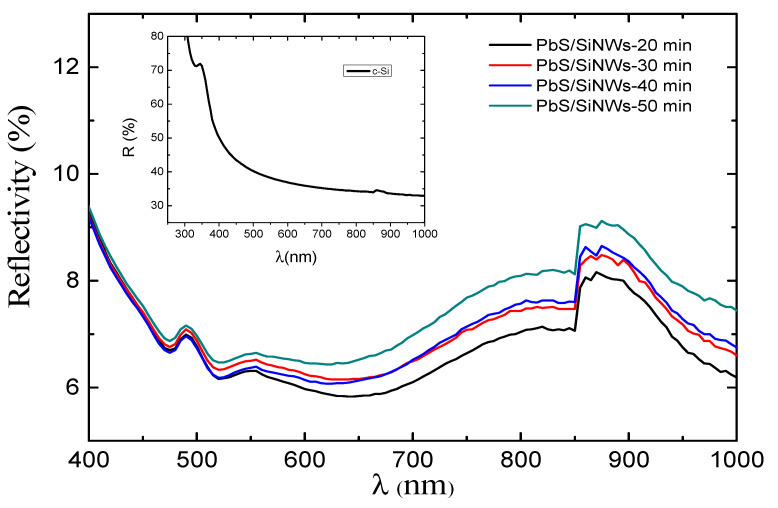
Reflectivity spectrum of a PbS NPs film (N_LP_ = 1000) deposited by PLD on different SiNWs substrates.

Figure 6 depicts the reflectivity of PbS/SiNWs nanohybrids versus etching time.

**Figure 7:**
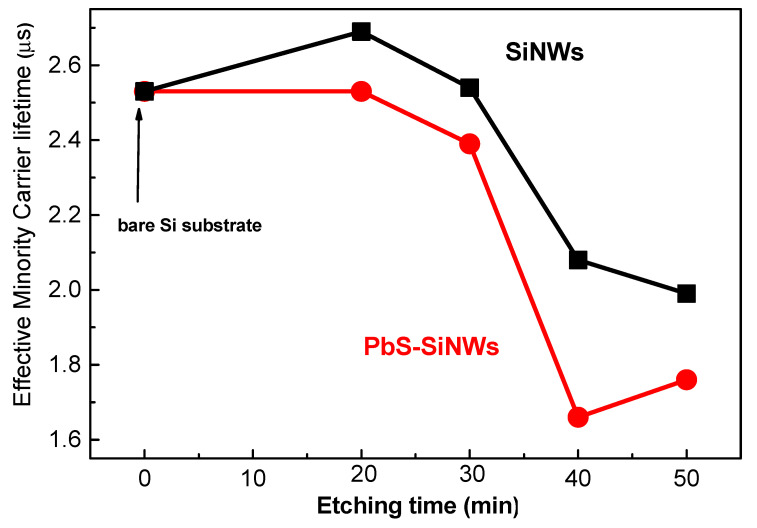
Effective minority carrier lifetime of Si substrate SiNWs/Si and PbS/SiNWs/Si measured at a carrier injection rate (Δn) equal to 2 × 10^14^ cm^−3^ for SiNWs etching time 20, 30, 40, and 50 min.

PbS/SiNWS nanohybrid reflectivity decreases sharply compared to the single-crystal Si substrate alone. The reflectivity minimum varies between 5.8 and 6.5% in the wavelength range 620–650 nm. The SiNWs seem to act as an effective anti-reflection coating due to light absorption increase due to the enlargement of the specific surface area. The latter recreates a significant role in photocatalysis, leading to increased catalytic activity, which will be demonstrated later. The Si wafer electronic properties (before and after treatment with PbS NPs) were quantified by measuring the effective minority carrier lifetime before and after forming the PbS/SiNWs nanohybrids. Figure 7 shows the evolution of the minority carrier lifetime (*τ_eff_*). If we assume that the Si bulk is not affected by the PbS/SiNWs surface treatment, only the minority carrier related to the surface of the Si substrate is concerned. The value of surface recombination rate (*S_eff_*) can be determined from the effective lifetime (*τ_eff_*) utilizing the below expression (2) [25,26]:

(2) $$\frac{1}{\tau_{eff}}=\frac{1}{\tau_{bulk}}+\frac{2S}{W}$$

where *τ_bulk_* and *W* are the lifetime of the bulk and the Si substrate thickness, respectively, if all samples are supposed to have relatively high values of *τ_bulk_* compared to *τ_S_*, the effective surface recombination is then reduced to Equation (3):

(3) $$\frac{1}{\tau_{eff}}=S_{eff}=\frac{W}{2\;\tau_{eff}}$$

The lifetime measurements are performed at a carrier injection rate (Δn) = 2 × 10^14^ cm^−3^. The effective lifetime value (*τ_eff_*) of the minority carriers of the untreated Si substrate is equal to 2.53 µs, and it decreases to 1.66 µs for SiNWs (50 min) decorated with PbS NPs. SiNWs present a sizeable internal surface full of defects; this may explain the degradation of the lifetime after the formation of the SiNWs. Some Ag-NPs residues can also affect the value of *τ_eff_* [27]. 

**Figure 8:**
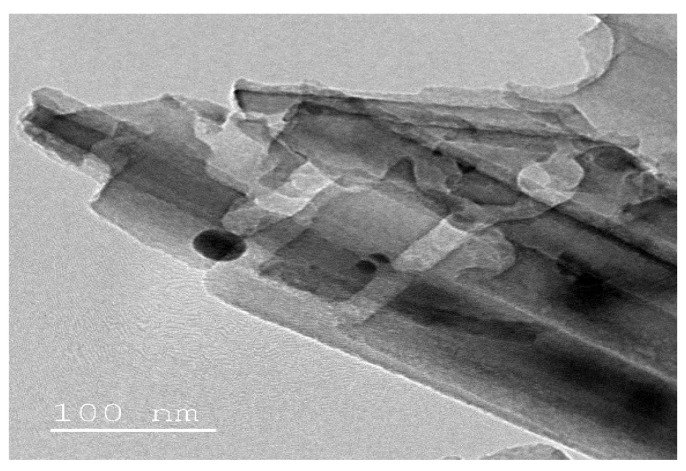
TEM image of the PbS/SiNWs nanohybrid.

For a better characterization of the nanostructural state of the PbS/SiNWs nanohybrid, transmission electron microscopy (TEM) observations were carried out on the prepared sample (Figure 8). The uniform morphology and size observed from Figure 8 are due to the ideal method used in this investigation. 

## 3. Photocatalytic Application of the PbS/SiNWs Nanohybrids

**Figure 9:**
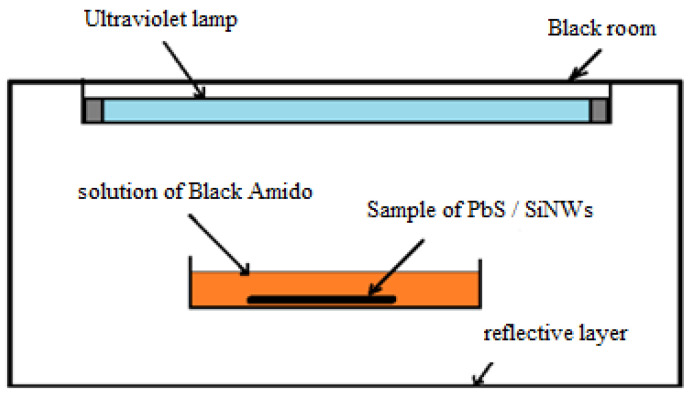
Photochemical reactor.

This experiment aims to study the efficacy of PbS/SiNWs nanohybrids for Photocatalysis application. The photochemical reactor is equipped with a 15 W ultraviolet lamp, as illustrated in Figure 9. The 1 × 1 cm^2^ PbS/SiNWs/Si substrate is immersed in 10 mL of aqueous Black Amido (BA) solution. 

**Figure 10:**
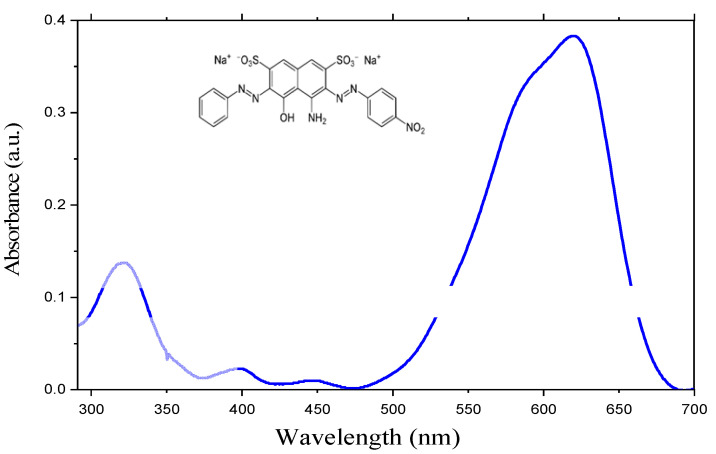
UV–Visible spectra of the BA-dye.

Then the dye solutions were exposed to UV irradiation at different times to reach the total degradation of the BA-dye. Later the solutions were analyzed using a UV-visible spectrophotometer. First, the effect of the SiNWs’ lengths (20, 30, 40 µm) on their photocatalytic performances while using the same BA-dye solution was investigated. Figure 9 presents the UV-visible spectra of the initial solution of Black Amido before photocatalysis. From Figure 10, a prominent peak at the ultraviolet region corresponding to a low energy π → π∗ transition of the aromatic benzene group can be observed [28,29]. Another spectral band peaking at 619 nm is due to the presence of the azo group. 

**Figure 11:**
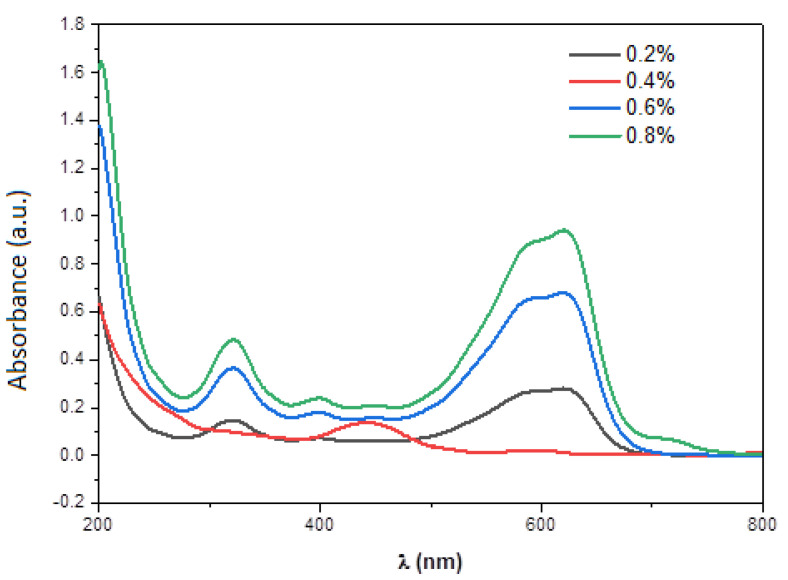
UV−Visible spectra of BA-dye for different volume concentration (0.2% 0.4% 0.6%, and 0.8%).

Figure 11 presents the UV-visible spectra for different volume concentration (0.2%, 0.4%, 0.6%, and 0.8%) of the Black Amido solution. After photocatalytic activity (Figure 10), we observed insignificant degradation for 0.2%, 0.6%, and 0.8% of BA-dye volume concentration. However, for 0.4% the peaks at 619 and 330 nm disappear revealing a complete degradation of the BA-dye in the solution. 

**Figure 12:**
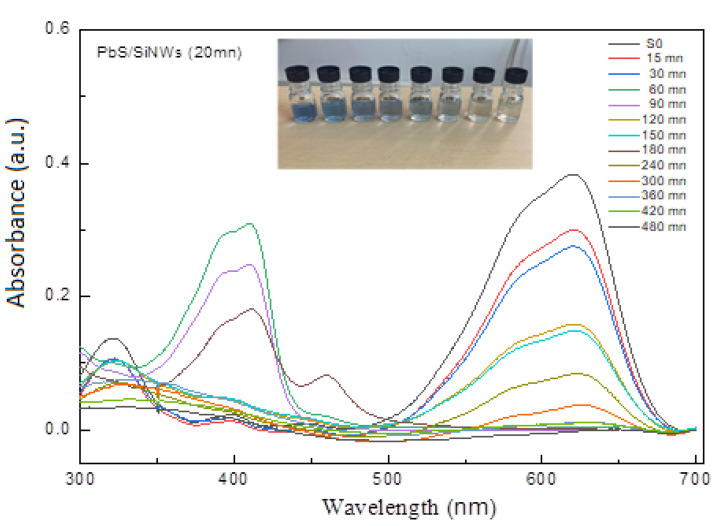
UV−visible spectra of BA-dye of volume concentration 0.4% in the presence of PbS/SiNWs nanohybrids (20 min) as a catalyst.

Figure 12 displays the UV-visible spectra of Black Amido solution in the presence of PbS/SiNWs for different degradation times. This figure illustrates a slight degradation effect during the 30 min UV exposure time accompanied by the PbS/SiNWs nanohybrids (20 min). However, starting from 4 h, the peaks at 619 and 330 nm decrease within UV exposure time, leading to a significant reduction in absorbance. A quasi-complete degradation of BA-dye is observed after 7 h. 

**Figure 13:**
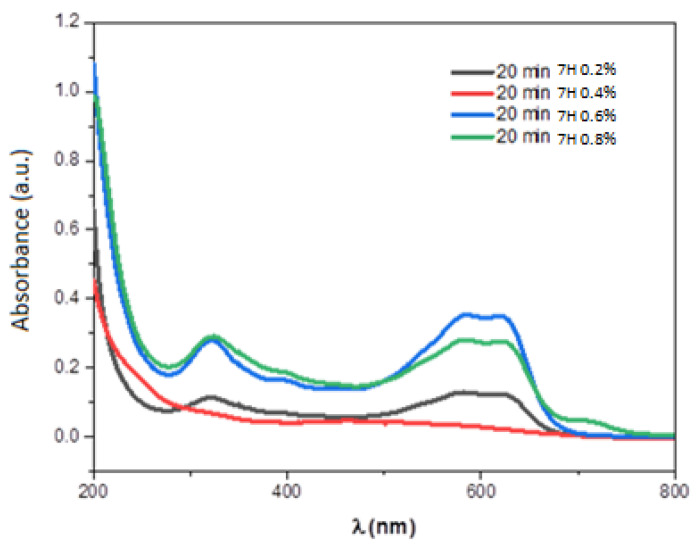
UV−Visible spectra of BA-dye in the presence of PbS/SiNWs nanohybrids (20 min) as a catalyst for different volume concentration (0.2% 0.4% 0.6%, and 0.8%).

The obtained results prove that the PbS/SiNWs are a good candidate for an efficient photocatalyst for wastewater treatment. In addition, the effect of PbS/SiNWs nanohybrids (20 min) on the photocatalytic activity for different volume concentrations of BA-dye was tested and shown in Figure 13. We noticed a complete degradation in the BA-dye for a volume concentration of 0.4% during 7 h exposure time. In fact, as the initial dye concentration rises, the number of adsorbed dye molecules on the surface of the PbS-SiNWs increases. Therefore, dye molecules occupy more and more active sites on the surface PbS-SiNWs catalyst. At a certain critical dye concentration most of active sites are occupied, leading to the degradation of most of dye. Beyond this critical concentration, active sites are saturated, and the un-adsorbed molecules control the photodegradation kinetics, thus reducing the reaction rate and then delaying the degradation process. 

**Figure 14:**
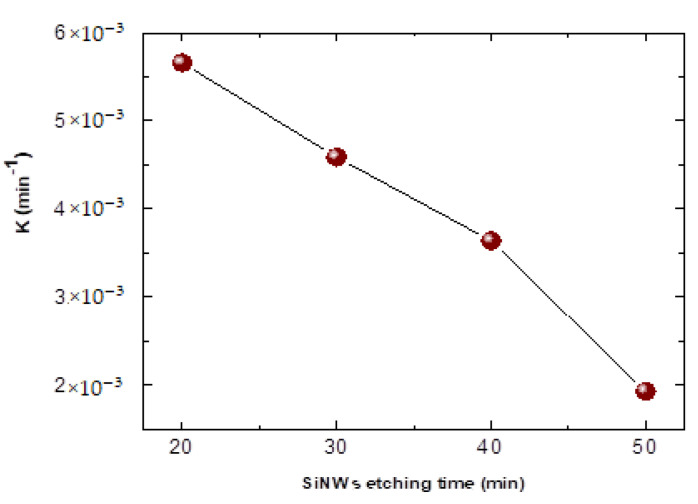
Kinetics curve of PbS/SiNWs sample for 20, 30, 40, and 50 min SiNWs etching time formation.

The effect of SiNWs length catalyst on the photocatalytic activity, degradation of a fixed concentration of BA-dye solution was carried out using 20, 30, 40, and 50 min of SiNWs etching time formation for a period of 7 h. It was found through this experiment that SiNWs of 20 min yields better photocatalytic degradation compared to other samples. This result may be due to the rise of the specific surface area, which plays a significant role in photocatalysis activity. Figure 14 depicts the evolution of the constant kinetic k of PbS/SiNWs (20, 30, 40, and 50 min). 

Figure 14 shows the PbS/SiNWs sample kinetics curve for 20, 30, 40, and 50 min SiNWs etching time formation. The reaction rate constant k is calculated using the first-order reaction rate constant. Knowing that the higher k is, the faster the reaction rate is. The results show that the reaction rate constant k of PbS/SiNWs sample for 20 is 5.7 × 10^−3^ min^−1^, which is 3 times higher than PbS/SiNWs sample for 50 (1.9 × 10^−3^ min^−1^) and 1.6 higher than PbS/SiNWs sample for 40 (3.5 × 10^−3^ min^−1^) and 1.3 higher than PbS/SiNWs sample for 30 (4.5 × 10^−3^ min^−1^).

## 4. Conclusions

This work investigated the effect of Si-NWs construction and their decoration using PbS nanoparticles on the optical, optoelectronic, and photodegradation properties. PbS-nanoparticles decorating SiNWs were applied for wastewater treatment. It has been attested that the PbS/SiNWs nanohybrids decrease the reflectivity, passivate the surface of the silicon substrate, and enhance the lifetime of the minority carriers. For photocatalytic application, the PbS/SiNWs nanohybrids exhibited promising photocatalytic activity. Almost total degradation of BA-dye was achieved in the presence of PbS/SiNWs nanohybrids catalyst under a UV irradiation period of 420 min. This result indicates that the PbS/SiNWs are an excellent candidate for the efficient degradation of organic dyes in wastewater.

## Figures and Tables

**Table 1:** Concentration of chemical compositions determined from the EDX spectrum.

Element	Line Type	Apparent Concentration	k Ratio	wt%	wt% Sigma	Atomic %
C	K series	5.71	0.05708	10.83	0.14	24.15
O	K series	7.19	0.06297	6.46	0.05	10.81
Si	K series	112.41	1.03086	61.64	0.25	58.77
S	K series	3.46	0.03667	2.39	0.07	2
Ag	L series	17.76	0.17758	15.61	0.21	3.88
Pb	M series	3.09	0.02972	3.07	0.26	0.4
Total				100		100

## Data Availability

Not applicable.
